# Optic nerve sonography in the diagnostic evaluation of adult brain injury

**DOI:** 10.1186/cc6897

**Published:** 2008-05-13

**Authors:** Theodoros Soldatos, Dimitrios Karakitsos, Katerina Chatzimichail, Matilda Papathanasiou, Athanasios Gouliamos, Andreas Karabinis

**Affiliations:** 1 Second Department of Radiology, Attikon University Hospital, 1 Rimin st, 124 62, Athens, Greece; 2 Department of Intensive Care, General State Hospital of Athens, 154 Mesogeion ave, 115 27, Athens, Greece

## Abstract

**Introduction:**

The optic nerve sheath diameter (ONSD) may be increased in brain-injured patients, especially children, with intracranial hypertension. We investigated whether measurements of ONSD correlated with simultaneous noninvasive and invasive measurements of the intracranial pressure (ICP) in brain-injured adults.

**Methods:**

Seventy-six critical care patients (58 males; 47 ± 18 years old) were included in the study. Fifty patients suffered from brain injury, whereas 26 had no intracranial pathology and served as control individuals. Initially, brain-injured patients were evaluated clinically (Glasgow Coma Scale) and using a semiquantitative (I to VI) neuroimaging scale (Marshall Scale). Thereafter, the patients were divided into those with moderate (Marshall Scale = I and Glasgow Coma Scale > 8 [*n *= 18]) and severe (Marshall Scale = II to VI and Glasgow Coma Scale ≤8 [*n *= 32]) brain injury. All patients underwent noninvasive measurement of the ICP (estimated ICP) by transcranial Doppler sonography, and synchronous ONSD measurements by optic nerve sonography. Finally, invasive ICP measurement using an intraparenchymal catheter was performed in patients with severe brain injury.

**Results:**

ONSD and estimated ICP were both significantly increased (6.1 ± 0.7 mm and 26.2 ± 8.7 mmHg, respectively; *P *< 0.0001) in patients with severe brain injury as compared with patients with moderate brain injury (4.2 ± 1.2 mm and 12.0 ± 3.6 mmHg) and compared with control individuals (3.6 ± 0.6 mm and 10.3 ± 3.1 mmHg). Furthermore, in patients with severe brain injury the ONSD measurements were strongly correlated with estimated ICP values (*r *= 0.80, *P *< 0.0001) as well as with the neuroimaging scale results (*r *= 0.82, *P *< 0.001). In the patients with severe brain injury, ONSD measurements correlated with invasive ICP values (*r *= 0.68, *P *= 0.002). The best cut-off value of ONSD for predicting elevated ICP was 5.7 mm (sensitivity = 74.1% and specificity = 100%).

**Conclusion:**

ONSD measurements correlate with noninvasive and invasive measurements of the ICP, and with head computed tomography scan findings in brain-injured adults. Hence, optic nerve sonography may serve as an additional diagnostic tool that could alert clinicians to the presence of elevated ICP, whenever invasive ICP evaluation is contraindicated and/or is not available. This trial is International Standard Randomised Controlled Trial Number registered (ISRCTN 91941687).

## Introduction

Elevated intracranial pressure (ICP) is a common manifestation of severe brain injury that requires rapid diagnosis and therapeutic intervention [1,2]. The use of an intracranial catheter remains the standard method for diagnosing intracranial hypertension [2-4], but this modality is not always feasible, either because contraindications such as coagulopathy or thrombocytopenia [5,6], or because of lack of neurosurgical expertise. Furthermore, noninvasive techniques for the evaluation of ICP have been developed and include computed tomography (CT) scan of the head, ophthalmoscopy and transcranial Doppler sonography (TCD). Unfortunately, each of these techniques has drawbacks. CT scan of the head is time consuming and requires transfer of critically ill patients and supporting devices to specialized facilities. Ophthalmoscopy necessitates experienced examiners and allows detection of elevated ICP but with some delay [7,8]. Finally, TCD may detect alterations of cerebral blood flow caused by elevated ICP [2,9-16] but also requires well trained observers, and inadequate sonic windows may prevent use of this technique in about 5% of cases [17,18].

Interestingly, the optic nerve sheath diameter (ONSD) was reported to be increased in patients with intracranial hypertension [1,4-7,19-27]. Previous reports have indicated that direct measurement of the ONSD is possible by optic nerve sonography and may be applied in brain-injured patient to detect elevated ICP [1,19,21]. In a previous study, we found that ONSD alterations were correlated with head CT scan results in brain-injured adults [19]. In the present study, we employed optic nerve sonography to investigate whether alterations in optic nerve diameter correlate with simultaneous noninvasive and invasive measurements of ICP in brain-injured adults. Noninvasive evaluation of the ICP was performed using TCD and invasive measurements of the ICP were performed by means of a surgically placed intracranial catheter.

## Materials and methods

### Patients

This study was performed from October 2006 to January 2008 in a cohort of 89 critical care patients, among which 62 suffered from brain injury whereas 27 had no intracranial pathology and served as control individuals. We excluded patients who were younger than 18 years, who had a history of glaucoma, or who had known disease of the optic nerve (inflammation or tumour). TCD was not technically feasible because of bilateral absence of the sonic window in four patients and one control individual; they were therefore excluded from the study. Also, eight patients with severe brain injury in whom invasive measurements of ICP were not performed were excluded from the study. Finally, 50 brain-injured patients and 26 control individuals were included in the statistical analysis.

In all participants mean arterial blood pressure (ABPm) was continuously monitored using an invasive arterial catheter in order to exclude hypotension (systolic blood pressure > 110 mmHg). Furthermore, heart rate was monitored in all participants to exclude bradycardia (heart rate < 60 beats/minute) and by pulse oximetry to exclude hypoxaemia (arterial oxygen saturation < 95%). In all mechanically ventilated patients (pressure-regulated volume control mode), arterial carbon dioxide tension was maintained at 33 to 35 mmHg throughout the study period. For all participants, the family's consent was obtained before their inclusion in the study. The study conformed with the principles outlined in the Declaration of Helsinki and was approved by the institutional ethics committee.

### Methods

Upon admission to the intensive care unit (ICU), all patients who had suffered head trauma underwent clinical evaluation (Glasgow Coma Scale [GCS]) by neurosurgeons, and consequently head CT scans were performed to evaluate possible brain injury. The CT scans were interpreted by experienced on-site radiologists. Based on the head CT scan results, the severity of brain injury was classified according to a semiquantitative neuroimaging scale (I to VI; Table 1), as described previously [19,28]. Based on the clinical and imaging findings, patients were divided into control individuals (group A), patients with moderate brain injury (Marshall Scale = I and GCS > 8; group B) and patients with severe brain injury (Marshall Scale = II to VI and GCS ≤8; group C).

**Table 1:** Classification of brain injury based on CT scan findings

Brain injury scale	CT scan findings
I	Normal CT scan (no visible pathology)
II	Cisterns present; midline shift 0 to 5 mm
III	Cisterns compressed or absent; midline shift 0 to 5 mm
IV	Midline shift > 5 mm
V	Any surgically evacuated mass lesion
VI	Lesion > 25 ml not surgically evacuated

CT, computed tomography.

Sonographic examinations were conducted using a Philips HD11XE (Philips Medical Systems; Bothell, WA, USA) equipped with a 2 MHz sector transducer and a 9 MHz linear transducer. All patients were examined in the supine position. TCD was performed in the middle cerebral arteries bilaterally, and the end-diastolic velocity (FVd) and mean velocity (FVm) were measured. For each side, an estimated ICP (eICP) value was calculated, using the following equation (as described previously [2]): eICP = ABPm × (1 - FVd/FVm) - 14. This equation for determining ICP noninvasively was tested in patients with brain injury and provided close approximations with invasively derived ICP values [2]. The eICP recorded was the average value obtained from repeated measurements of both sides, which were taken once an hour over 10 working hours for 2 days after admission. In those patients with a unilateral absent sonic window or in whom it was not possible to scan one side because of surgical wounds, only measurements from the contralateral side were recorded.

**Figure 1:**
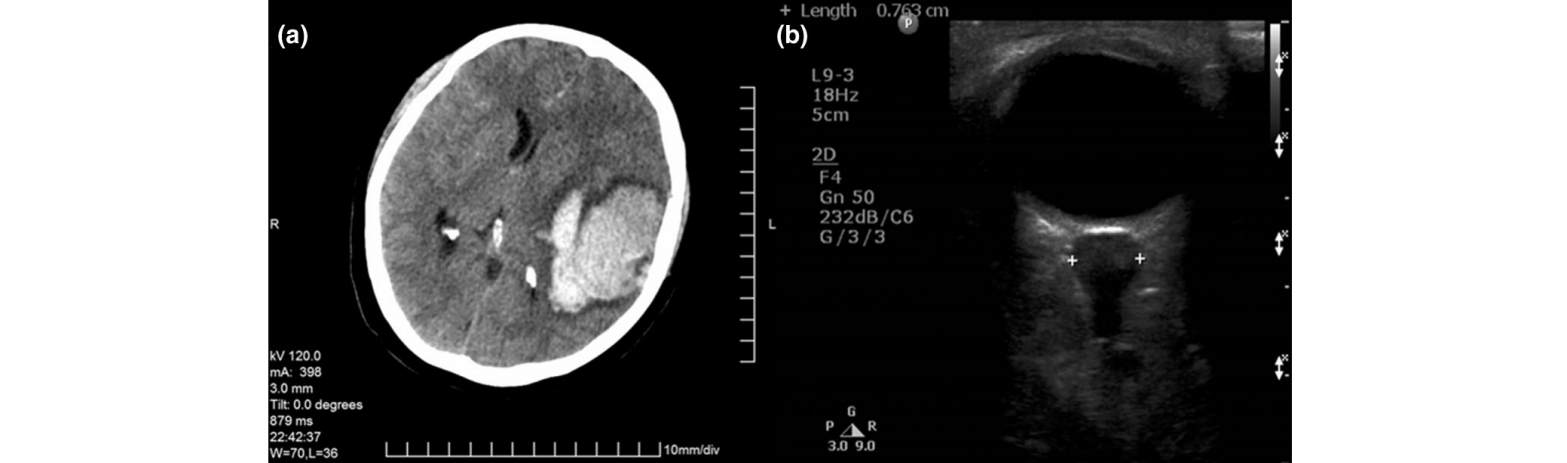
Imaging findings of a brain-injured adult. **(a) **Brain computed tomography (CT) scan of a patient showing a midline shift of more than 5 mm and a nonevacuated lesion of more than 25 ml. **(b) **Transorbital sonography of the same patient documenting increased optic nerve sheath diameter.

During the same sonographic session, immediately after TCD measurements, optic nerve studies were performed. The ONSD was measured 3 mm posterior to the papilla, as described previously [1,4-8,19-27,29] (Figure 1). The ONSD recorded was the average value obtained from repeated measurements in both eyes, which were performed once an hour over 10 working hours for 2 days after admission. In patients with orbitofacial trauma and in whom it was not possible to scan both eyes, only the unaffected eye was examined and the resulting ONSD was recorded.

TCD and ocular sonography were performed by experienced observers who were blinded to patient identity and to invasive ICP findings. Furthermore, the difference from the mean value for each eye of each single measurement for each observer was calculated. Median intra-observer variation was then calculated for the total study population. Median values for the differences between the mean values from each observer (inter-observer variation) were also determined [30]. Finally, in patients with severe brain injury and concomitant cerebral oedema (group C), a Camino intraparenchymal catheter (Camino Laboratories, San Diego, CA, USA) was inserted by neurosurgeons in the frontal region for a period of 7 days. During the above sonographic sessions, the ICP measurements were simultaneously taken once an hour over 10 working hours for 2 days after admission. Hence, the average value of the ICP measurements, which were electronically recorded, was finally included in the statistical analysis. Elevated ICP was defined as an ICP of 20 mmHg or greater [3].

### Statistical analysis

Summary data are expressed as mean ± standard deviation. Student's *t*-test for independent samples was used to compare the mean ONSD values and mean eICP values between various groups. Pair-wise multiple comparisons were performed using the Tukey critical difference method. Wilcoxon matched-pairs test was employed to determine differences between ICP and eICP measurements. Correlations between continuous variables were assessed using the Pearson correlation coefficient. For ordinal data the Spearman rank correlation was used. A two-tailed significance level of 0.05 was regarded statistically significant. In addition, receiver operating characteristic (ROC) curves were obtained to specify cut-off values of ONSD and eICP for the prediction of elevated ICP. Cut-off values were the threshold values that maximized the sum of specificity and sensitivity. All data were stored on a spreadsheet (Excel 2003; Microsoft, Seattle, WA, USA), and analyses were performed using a commercially available statistical package (MedCalc 8.0; MedCalc Software, Mariakerke, Belgium).

## Results

The characteristics of the study population are presented in Table 2. There were no significant differences in age, sex and body mass index between patient groups. The mean period of hospitalization in all head injured patients was 45 ± 38 days. All brain CT scans were performed upon admission to the ICU. During the first 48 hours after admission, all patients underwent ONSD measurements as well as noninvasive ICP measurements. The average values of the these measurements were used in the statistical analyses. The median intra-observer variation for ONSD was 0.2 mm (95% confidence interval [CI] = 0.1 mm to 0.5 mm). The median inter-observer variation of ONSD was 0.3 mm (95% CI = 0.1 mm to 0.7 mm).

**Table 2:** Characteristics of the study population

Characteristic	Group A (control individuals; *n *= 26)	Group B (moderate brain injury; *n *= 18)	Group C (severe brain injury; *n *= 32)
Age (years; mean ± SD)	49 ± 19	43 ± 14	49 ± 18
Sex (male/female; *n*)	21/5	12/6	25/7
BMI (kg/m^2^; mean ± SD)	20 ± 4.9	20 ± 4.5	19.2 ± 6.0
GCS (1 to 14; median [range])	14	11.1 (8 to 13)	4.9 (3 to 7)*
Diagnosis upon admission (*n *[%])	Trauma: 11 (42%)	Head injury: 18 (100%)	Head injury: 32 (100%)
	Sepsis: 8 (31%)		
	ARDS: 5 (19%)		
	Burn: 2 (8%)		
ONSD (mm; mean ± SD [range])	3.6 ± 0.6 (2.2 to 4.9)	4.2 ± 1.2 (3.0 to 6.2)	6.1 ± 0.7 (5.2 to 7.8)*
eICP (mmHg; mean ± SD [range])	10.3 ± 3.1 (3.5 to 14.7)	12.0 ± 3.6 (6.3 to 18.4)	26.2 ± 8.7 (14.4 to 51.1)^†^
ICP (mmHg; mean ± SD [range])	-	-	26.0 ± 7.3 (16.0 to 47.0)
CPP (mmHg; mean ± SD [range])	-	-	61.8 ± 12.0 (29.0 to 85.0)
Brain CT injury scale upon admission (I to VI; *n *[%])	Normal	I: 18 (100%)	II: 4 (12.5%)
			III: 16 (50.0%)
			IV: 4 (12.5%)
			V: 4 (12.5%)
			VI: 4 (12.5%)

*Statistically significant difference in GCS and ONSD values between group C and groups A and B (*P *< 0.0001; Student's *t*-test). ^†^Statistically significant difference in eICP values between group C and groups A and B (*P *< 0.0001; Student's *t*-test). ARDS, adult respiratory distress syndrome; BMI, body mass index; CPP, cerebral perfusion pressure; CT, computed tomography; eICP, non-invasive intracranial pressure; GCS, Glasgow Coma Scale; ICP, invasive intracranial pressure; ONSD, optic nerve sheath diameter; SD, standard deviation.

A Camino intraparenchymal catheter was inserted in all patients with severe brain injury. Bilateral TCD examination was performed in 71 patients and unilateral TCD examination in five patients because of technical difficulties. Seventy-four patients underwent bilateral optic nerve examination, whereas two patients underwent only unilateral examination because of orbitofacial trauma.

**Figure 2:**
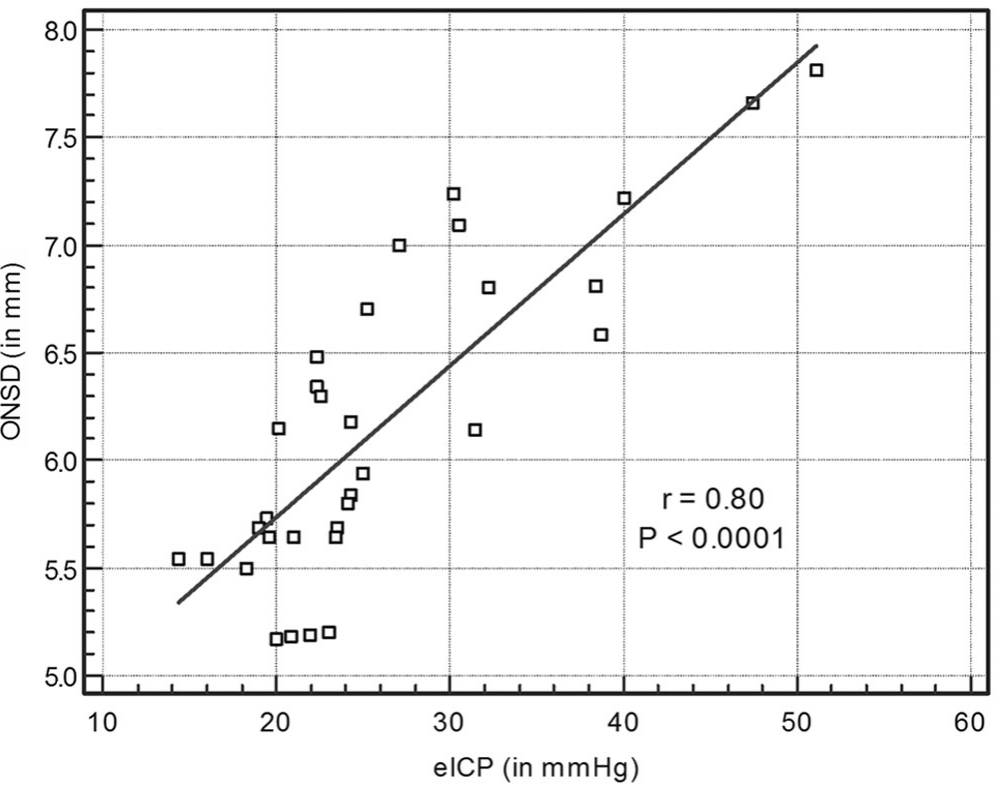
ONSD versus eICP. Shown are the optic nerve sheath diameter (ONSD) measurements plotted against the noninvasive intracranial pressure (eICP) in patients with severe brain injury (group C; *n *= 32).

There were no significant differences in ONSD and eICP measurements between groups A and B (Table 2). The ONSD and eICP in group C were significantly increased as compared with those in groups A and group B (*P *< 0.0001; Table 2). In group C, a significant correlation was found between ONSD and eICP (*r *= 0.80, 95% CI = 0.62 to 0.90; *P *< 0.0001; Figure 2) and between the ONSD values and the neuroimaging scale findings (*r *= 0.82, 95% CI = 0.66 to 0.91; *P *< 0.0001).

**Figure 3:**
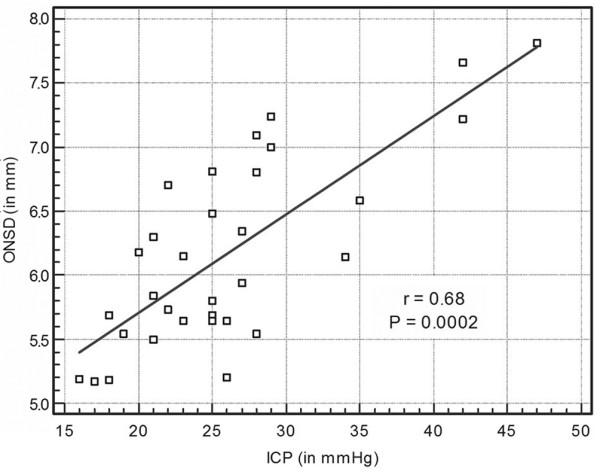
ONSD versus invasive ICP. Shown are the optic nerve sheath diameter (ONSD) measurements plotted versus the invasive intracranial pressure (ICP) in the patients with severe brain injury (group C; *n *= 32).

It is of note that five patients (group C) underwent neurosurgical interventions to evacuate mass lesions. Despite prompt surgical and medical therapeutic interventions, 11 patients (group C) progressed toward brain tamponade. In our study, ONSD measurements did not correlate significantly with eICP measurements in control individuals (group A; *r *= 0.29, *P *= 0.15) and in patients with moderate brain injury (group B; *r *= 0.32, *P *= 0.11). In group C patients invasive ICP correlated both with ONSD (*r *= 0.68, 95% CI = 0.43 to 0.83; *P *= 0.002; Figure 3) and with eICP (*r *= 0.63, 95% CI = 0.36 to 0.80; *P *= 0.0005).

**Figure 4:**
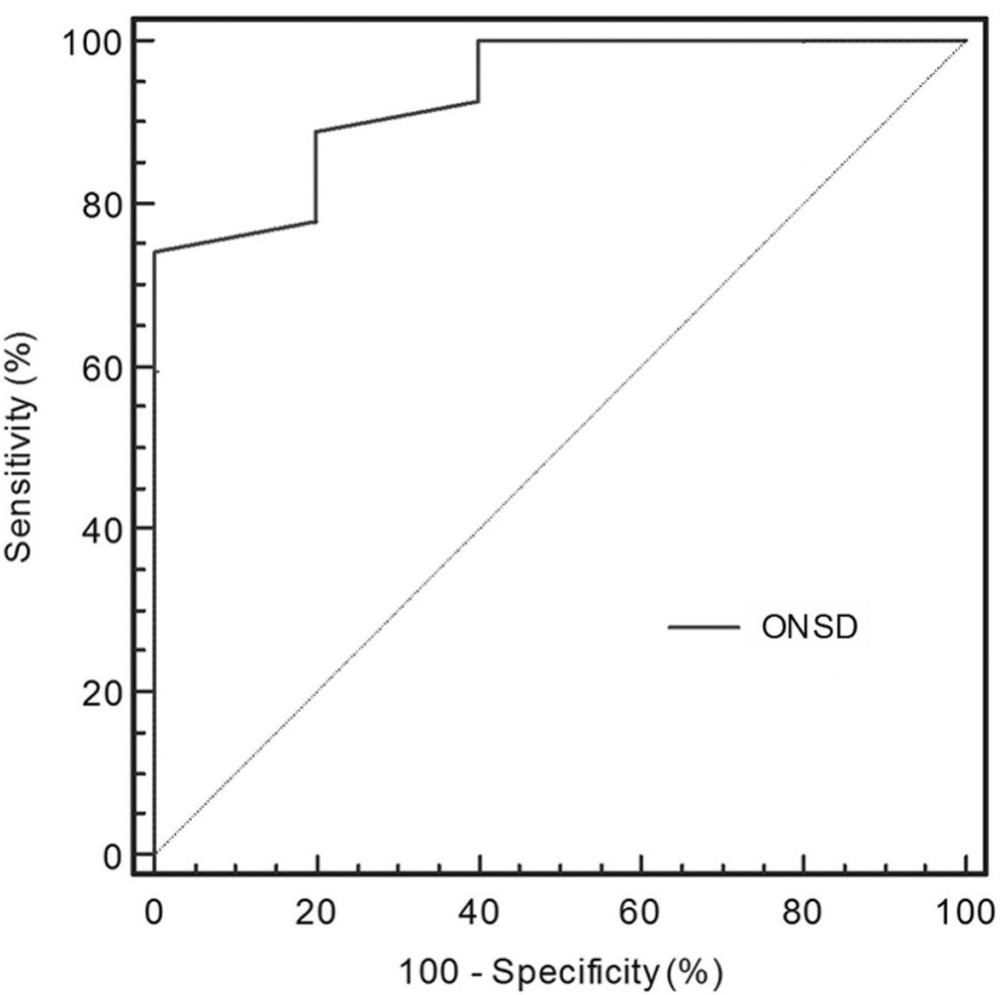
Predictive value of ONSD. Presented is a receiver operating characteristic curve showing the predictive value of the optic nerve sheath diameter (ONSD; the cut-off value is 5.7 mm) for elevated intracranial pressure (ICP; ≥20 mmHg).

The ROC curve results revealed that the optimal cut-off value of ONSD for predicting elevated ICP was 5.7 mm (area under the ROC curve = 0.93, 95% CI = 0.79 to 0.99; Figure 4). The sensitivity and the specificity of this cut-off value were 74.1% and 100%, respectively.

## Discussion

In brain-injured adults elevated ICP and concomitant brain oedema may reduce cerebral perfusion pressure and oxygen delivery to the brain, thus promoting ischaemia and progression toward brain tamponade [2,31]. Despite their clinical and technical limitations, TCD and optic nerve sonography are becoming increasingly popular as additional neuromonitoring tools in the ICU. They are both noninvasive and easily accessible modalities, which could be used for rapid bedside evaluation of patients with head trauma [1-3,19,21,31-33]. In a previous report [19] we established the strong correlation between ONSD measurements and brain CT scan results in brain-injured adults. However, in this report the brain CT scan was mainly used upon admission in order to assist clinicians with classifying the severity of brain injury. In this study, we employed optic nerve sonography to investigate whether the ONSD measurements correlated with noninvasive and invasive ICP measurements in brain-injured patients.

In agreement with previous studies, we found that patients with severe brain injury had increased ONSD as compared with control individuals and/or patients with moderate brain injury [1,19,21]. We also found that patients with severe brain injury exhibited increased values of eICP, as estimated by means of TCD; this is in accordance with previous reports [2,9,10]. However, the estimation of ICP dynamics and/or cerebral blood flow by means of TCD is rather an approximation, because these are influenced by complex autoregulatory mechanisms, such as dilatation or constriction of cerebral vessels, which cause changes in intracranial volume [16]. Nevertheless, we employed the equation introduced by Csoznyka and coworkers [2]: eICP = ABPm × (1 - FVd/FVm) - 14. The present findings indicate that in patients with severe brain injury, invasive and noninvasive ICP values correlated significantly. However, they were significantly different from identical, which is in accordance with previous reports [2].

Interestingly, brain oedema may hinder the insertion of an invasive device or may even impede free circulation of cerebrospinal fluid (CSF) in the ventricular system, rendering global ICP measurements questionable [2,3]. This by no means suggests that TCD is a superior method in estimating ICP. It is rather a measure that provides a respective 'global' assessment of the real-time ICP, as measured invasively. Hence, the intracranial catheter remains the standard method with which to identify intracranial hypertension. Furthermore, when Czosnyka and coworkers [2] compared noninvasive ICP findings provided by their equation with ICP measurements obtained using an intraparenchymal probe, they reported an error of less than 5 mmHg in only 39% of the measurements. They interpreted this error as because due either to the inherent inaccuracy of the noninvasive ICP measurement or to the uneven distribution of CSF in cases of brain trauma and midline shift, in which the ICP of the intraparenchymal probe may differ from that affecting the blood flow in the area of the middle cerebral artery.

Another interesting finding of the present study is that ONSD measurements were significantly correlated with both invasive and noninvasive measurements of ICP. Geeraerts and coworkers [21] evaluated brain-injured patients and observed a strong correlation between ONSD and invasive ICP [21]. Gangemi and colleagues [22] examined patients with intracranial hypertension syndromes of various aetiology and observed a gradual postoperative decrease in the initially abnormal ONSD until the latter reached the normal values. Hansen and coworkers [29] investigated the optic nerve sheath response to pressure during CSF absorption studies in patients undergoing neurological testing and observed a sheath enlargement, which was completely reversible in all individuals during the infusion tests, as well as a linear covariance between ONSD and CSF pressure. However, in our study ONSD measurements did not correlate with eICP measurements in control individuals and in patients with moderate brain injury, indicating that the optic nerve sheath has a baseline diameter that remains constant as long as ICP is maintained within normal limits.

The enlargement of the optic nerve sheath is believed to underlie the equilibration of CSF pressure between orbital and cranial cavities. In cases of elevated ICP, CSF flows toward the perineural subarachnoid space, increasing the pressure around the optic nerve and expanding the optic nerve sheath [29]. Although it is difficult to suggest a precise cut-off value, most authors have suggested that the upper normal value of ONSD ranges between 4.5 and 5 mm, and that ONSD values greater than this threshold should prompt suspicion of elevated ICP [1,4,20,24,27,29,34]. Based on our data, the best cut-off ONSD value for predicting elevated ICP was 5.7 mm, which is very close to the cut-off point recently suggested by Geeraerts and colleagues [21]. Finally, the present data confirmed the high intra-observer and inter-observer reproducibility of optic nerve sonography, as previously reported [19,35-37].

### Study limitations

In a previous study we found that monitoring of the ONSD in patients with severe brain injury had no significant prognostic value for final outcome [19]. Also, ONSD measurements should be carefully evaluated by clinicians. Optic nerve sheath enlargement can also occur because of secondary involvement as a result of a variety of orbital and systematic abnormalities, such as tumour, inflammation, Grave's disease, sarcoidosis, pseudotumour, metastasis, haemorrhage in and around the optic nerve complex, and hydrops from extrinsic tumour [38]. Additionally, orbital fractures and optic nerve injury may occur in up to 10% of patients with head trauma [39]. The effect of these injuries on the sonographic depiction of the optic nerve and on the ONSD remains unclear [21].

Optic nerve sonography and TCD both have technical limitations and require a high level of expertise. ONSD measures are very small, and any alterations approach the intrinsic accuracy limits of the ultrasound equipment [35]. The absence of sonic window renders TCD impossible to perform in approximately 5% of patients [17,18]. Finally, both methods may be impossible to perform because of superficial surgical wounds or severe anatomic alterations in patients with head trauma. In the present study we found that ONSD measurements could provide information useful for determining the severity of adult brain injury. However, larger studies are clearly required to establish optic nerve sonography as a standard method in the diagnostic evaluation of adult brain injury. Furthermore, whether optic nerve sonography enables monitoring of changes in ICP over time remains to be clarified.

## Conclusion

ONSD measurements correlate with noninvasive and invasive measurements of ICP as well as with head CT scan findings in brain-injured adults. The above measurements may provide useful information regarding the presence of cerebral oedema and intracranial hypertension, and could therefore be applied in the ICU setting to the diagnostic evaluation of adult brain injury. Hence, optic nerve sonography may serve as an additional diagnostic tool, which could alert clinicians to the presence of elevated ICP, whenever invasive ICP evaluation is contraindicated and/or is not available.

## Key messages

• ONSD measurements correlate with noninvasive and invasive measurements of the ICP, as well as with head CT scan findings in brain injured adults.

• ONSD measurements may provide useful information regarding the presence of cerebral oedema and intracranial hypertension, and could therefore be applied in the ICU setting to the diagnostic evaluation of adult brain injury.

• The optic nerve sheath has a baseline diameter that remains constant as long as ICP is maintained within normal limits.

• Optic nerve sonography may serve as an additional diagnostic tool, which could alert clinicians to the presence of elevated ICP, whenever invasive ICP evaluation is contraindicated and/or is not available.

## Abbreviations

CI = confidence interval; CSF = cerebrospinal fluid; CT = computed tomography; FVd = end-diastolic velocity; FVm = mean velocity; eICP = estimated intracranial pressure; GCS = Glasgow Coma Scale; ISU = intensive care unit; ICP = intracranial pressure; ONSD = optic nerve sheath diameter; ROC = receiver operating characteristic; TCD = transcranial Doppler sonography.

## Competing interests

The authors declare that they have no competing interests.

## Authors' contributions

TS and DK designed the study, performed optic nerve and TCD examinations in the ICU setting, performed the statistical analysis and drafted the manuscript. KC, MP and AG interpreted the CT scans, provided expert advice concerning the design of the study, and contributed to the statistical analysis. AK participated in the design of the study and provided overall guidance. All authors read and approved the final manuscript.

## References

[B1] Tayal VS, Neulander M, Norton HJ, Foster T, Saunders T, Blaivas M (2007). Emergency department sonographic measurement of optic nerve sheath diameter to detect findings of increased intracranial pressure in adult head injury patients. Ann Emerg Med.

[B2] Czosnyka M, Matta BF, Smielewski P, Kirkpatrick PJ, Pickard JD (1998). Cerebral perfusion pressure in head-injured patients: a noninvasive assessment using transcranial Doppler ultrasonography. J Neurosurg.

[B3] Czosnyka M, Pickard JD (2004). Monitoring and interpretation of intracranial pressure. J Neurol Neurosurg Psychiatry.

[B4] Tsung JW, Blaivas M, Cooper A, Levick NR (2005). A rapid noninvasive method of detecting elevated intracranial pressure using bedside ocular ultrasound: application to 3 cases of head trauma in the pediatric emergency department. Pediatr Emerg Care.

[B5] Seestedt RC, Frankel MR (1999). Intracerebral hemorrhage. Curr Treat Options Neurol.

[B6] Lee LA, Sharar SR, Lam AM (2002). Perioperative head injury management in the multiply injured trauma patient. Int Anesthesiol Clin.

[B7] Malayeri AA, Bavarian S, Mehdizadeh M (2005). Sonographic evaluation of optic nerve diameter in children with raised intracranial pressure. J Ultrasound Med.

[B8] Helmke H, Hansen HC (1996). Fundamentals of transorbital sonographic evaluation of optic nerve sheath expansion under intracranial hypertension. II. Patient study. Pediatr Radiol.

[B9] Bellner J, Romner B, Reinstrup P, Kristiansson KA, Ryding E, Brandt L (2004). Transcranial Doppler sonography pulsatility index (PI) reflects intracranial pressure (ICP). Surg Neurol.

[B10] Voulgaris SG, Partheni M, Kaliora H, Haftouras N, Pessach IS, Polyzoidis KS (2005). Early cerebral monitoring using the transcranial Doppler pulsatility index in patients with severe brain trauma. Med Sci Monit.

[B11] Splavski B, Radanović B, Muzević D, Has B, Janculjak D, Kristek J, Jukić D (2006). Assessment of intra-cranial pressure after severe traumatic brain injury by transcranial Doppler ultrasonography. Brain Inj.

[B12] Schmidt B, Czosnyka M, Klingelhöfer J (2002). Clinical applications of a non-invasive ICP monitoring method. Eur J Ultrasound.

[B13] Homburg AM, Jakobsen M, Enevoldsen E (1993). Transcranial Doppler recordings in raised intracranial pressure. Acta Neurol Scand.

[B14] Chan KH, Miller JD, Dearden NM, Andrews PJ, Midgley SL (1992). The effect of changes in cerebral perfusion pressure upon middle cerebral artery blood flow velocity and jugular bulb venous oxygen saturation after severe brain injury. J Neurosurg.

[B15] Klingelhöfer J, Conrad B, Benecke R, Sander D, Markakis E (1988). Evaluation of intracranial pressure from transcranial Doppler studies in cerebral disease. J Neurol.

[B16] Schmidt B, Czosnyka M, Raabe A, Yahya H, Schwarze JJ, Sackerer D, Sander D, Klingelhöfer J (2003). Adaptive noninvasive assessment of intracranial pressure and cerebral autoregulation. Stroke.

[B17] Hassler W, Steinmetz H, Gawlowski J (1988). Transcranial Doppler ultrasonography in raised intracranial pressure and in intracranial circulatory arrest. J Neurosurg.

[B18] Aaslid R, Huber P, Nornes H (1986). A transcranial Doppler method in the evaluation of cerebrovascular spasm. Neuroradiology.

[B19] Karakitsos D, Soldatos T, Gouliamos A, Armaganidis A, Poularas J, Kalogeromitros A, Boletis J, Kostakis A, Karabinis A (2006). Transorbital sonographic monitoring of optic nerve diameter in patients with severe brain injury. Transplant Proc.

[B20] Newman WD, Hollman AS, Dutton GN, Carachi R (2002). Measurement of optic nerve sheath diameter by ultrasound: a means of detecting acute raised intracranial pressure in hydrocephalus. Br J Opthalmol.

[B21] Geeraerts T, Launey Y, Martin L, Pottecher J, Vigué B, Duranteau J, Benhamou D (2007). Ultrasonography of the optic nerve sheath may be useful for detecting raised intracranial pressure after severe brain injury. Intensive Care Med.

[B22] Gangemi M, Cennamo G, Maiuri F, D'Andrea F (1987). Echographic measurement of the optic nerve in patients with intracranial hypertension. Neurochirurgia (Stuttg).

[B23] Brzezinska R, Schumacher R (2002). Diagnosis of elevated intracranial pressure in children with shunt under special consideration of transglobe sonography of the optic nerve. Ultraschall Med.

[B24] Blaivas M, Theodoro D, Sierzenski PR (2005). Elevated intracranial pressure detected by bedside emergency ultrasonography of optic nerve sheath. Acad Emerg Med.

[B25] Galetta S, Byrne SF, Smith JL (1989). Echographic correlation of optic nerve sheath size and cerebrospinal fluid pressure. J Clin Neuroopthalmol.

[B26] Helmke K, Burdelski M, Hansen HC (2000). Detection and monitoring of intracranial pressure dysregulation in liver failure by ultrasound. Transplantation.

[B27] Girisgin AS, Kalkan E, Kocak S, Cander B, Gul M, Seniz M (2007). The role of optic nerve ultrasonography in the diagnosis of elevated intracranial pressure. Emerg Med J.

[B28] Marshall LF, Marshall SB, Klauber MR, Van Berkum Clark M, Eisenberg H, Jane JA, Luerssen TG, Marmarou A, Foulkes MA (1992). The diagnosis of head injury requires a classification based on computed axial tomography. J Neurotrauma.

[B29] Hansen HC, Helmke K (1997). Validation of the optic nerve sheath response to changing cerebrospinal fluid pressure: ultrasound findings during intrathecal infusion tests. J Neurosurg.

[B30] Bland JM, Altman DG (1994). Correlation, regression, and repeated data. BMJ.

[B31] Kirkpatrick PJ, Chan KH, Reilly P, Bullock R (1997). Transcranial Doppler. Head injury.

[B32] Gupta AK (2002). Monitoring the injured brain in the intensive care unit. J Postgrad Med.

[B33] Bhatia A, Gupta AK (2007). Neuromonitoring in the intensive care unit. I. Intracranial pressure and cerebral blood flow monitoring. Intensive Care Med.

[B34] Romagnuolo L, Tayal V, Tomaszewski C, Saunders T, Norton HJ (2005). Optic nerve sheath diameter does not change with patient position. Am J Emerg Med.

[B35] Ballantyne SA, O'Neill G, Hamilton R, Hollman AS (2002). Observer variation in the sonographic measurement of optic nerve sheath diameter in normal adults. Eur J Ultrasound.

[B36] Helmke H, Hansen HC (1996). Fundamentals of transorbital sonographic evaluation of optic nerve sheath expansion under intracranial hypertension. I. Experimental study. Pediatr Radiol.

[B37] Beatty S, Good PA, McLaughlin J, O'Neill EC (1998). Echographic measurements of the retrobulbar optic nerve in normal and glaucomatous eyes. Br J Opthalmol.

[B38] Rothman MI, Zoarski GH, Sutton D (2003). The orbit. Textbook of Radiology and Imaging.

[B39] Holt GR, Holt JE (1983). Incidence of eye injuries in facial fractures: an analysis of 727 cases. Otolaryngol Head Neck Surg.

